# Psychosocial rehabilitation for severe mental illness: A community project adjustable to the needs and resources of the population

**DOI:** 10.1192/j.eurpsy.2021.1345

**Published:** 2021-08-13

**Authors:** S. Freitas Ramos, G. Farelo, M. Moura, M. Araújo, S. Carvalho, F. Ferreira, R. Quelhas

**Affiliations:** 1 Department Of Psychiatry And Mental Health, Local Health Unit of Guarda, Guarda, Portugal; 2 Mental Health Department, Hospital Pedro Hispano, Matosinhos Local Health Unit, Matosinhos, Portugal; 3 Porto School Of Education, Porto Polytechnic, Porto, Portugal; 4 Ciap, Centro Incentivar a Partilha, Matosinhos, Portugal

**Keywords:** solidarity, community mental health, Rehabilitation

## Abstract

**Introduction:**

Psychosocial rehabilitation is a challenge in a society with demands unsuitable for those with severe mental illness (SMI). The Mental Health Department of Matosinhos Local Health Unity (MHD-MLHU) has developed a solidarity project aiming to evaluate and elaborate individualized rehabilitative responses with people with SMI, including people from the community motivated for solidarity initiatives.

**Objectives:**

To describe a psychosocial rehabilitation project focused on community integration of people with SMI, considering needs and resources of the population, and to present the individualized rehabilitation plans carried out for people with SMI.

**Methods:**

In January 2019, we began the home evaluation of people with SMI monitored in the MHD-MLHU. To develop solidary based play-occupational groups, we interviewed people from the community and from the common mental pathology outpatient clinic willing to participate.

**Results:**

We present the description and evaluation of the psychosocial responses developed by the project. These responses include recreational-occupational groups, tailored to interests of each person with SMI, and using the community support group built for the purpose. These responses promote face-to-face activities, and enhance the destigmatization of SMI. The constraints resulting from the COVID-19 pandemic led to the creation of digital responses aimed at people with SMI and the community in general.

**Conclusions:**

This experience has revealed the great potential of rehabilitating the community context of people with SMI, rather than just contemplating pre-existing structured responses. The pandemic created specific challenges but made the initiative even more relevant for SMI people and for promoting the mental health of the general population.

